# The Activity of Spontaneous Action Potentials in Developing Hair Cells Is Regulated by Ca^2+^-Dependence of a Transient K^+^ Current

**DOI:** 10.1371/journal.pone.0029005

**Published:** 2011-12-22

**Authors:** Snezana Levic, Ping Lv, Ebenezer N. Yamoah

**Affiliations:** 1 Program in Communication Science, Department of Anesthesiology and Pain Medicine, School of Medicine, University of California Davis, Davis, California; 2 Department of Pharmacology, Hebei Medical University, Shijiazhuang, China; University of Houston, United States of America

## Abstract

Spontaneous action potentials have been described in developing sensory systems. These rhythmic activities may have instructional roles for the functional development of synaptic connections. The importance of spontaneous action potentials in the developing auditory system is underpinned by the stark correlation between the time of auditory system functional maturity, and the cessation of spontaneous action potentials. A prominent K^+^ current that regulates patterning of action potentials is I_A_. This current undergoes marked changes in expression during chicken hair cell development. Although the properties of I_A_ are not normally classified as Ca^2+^-dependent, we demonstrate that throughout the development of chicken hair cells, I_A_ is greatly reduced by acute alterations of intracellular Ca^2+^. As determinants of spike timing and firing frequency, intracellular Ca^2+^ buffers shift the activation and inactivation properties of the current to more positive potentials. Our findings provide evidence to demonstrate that the kinetics and functional expression of I_A_ are tightly regulated by intracellular Ca^2+^. Such feedback mechanism between the functional expression of I_A_ and intracellular Ca^2+^ may shape the activity of spontaneous action potentials, thus potentially sculpting synaptic connections in an activity-dependent manner in the developing cochlea.

## Introduction

Structured spontaneous action potentials (SAPs) play an instructive role in the survival and refinement of neuronal connections in developing sensory systems [1]–[5]. Indeed, SAPs with different firing patterns encode information that may translate into variable gene expression that contributes to proper functional development [3], [6]–[8]. Similarly, developing hair cells (HCs) fire SAPs before the onset of hearing [9]–[14], at a period when major synaptic refinement occurs within the cochlea and cochlear nucleus (CN) [9], [13], [15]–[17]. Before the onset of hearing, auditory neurons at various levels of the auditory pathway show low, bursting and occasional rhythmic spontaneous activity. This rhythmic activity is robust in the cochlear ganglion cells of the pre-hatched chickens [18], [19], and the signal is abolished at higher levels by silencing the cochlea with the tetrodotoxin (TTX) injections or cochlear ablation [20]. Thus, SAPs may arise in the cochlea and it has been predicted that they may be modulated by the release of ATP from supporting cells and potentially contribute towards the establishment of proper tonotopic maps along auditory axes [21]–[23]. Incidentally, SAPs reappear during HC regeneration in chicken basilar papilla [9].

Spontaneous activity in developing HCs is Ca^2+^-dependent [9], [13], [16], [23], producing rhythmic changes in intracellular Ca^2+^ (Ca^2+^_i_) [24], [25]. Moreover, phasic alterations of Ca^2+^_i_ mediate the expression of K^+^ channels that modulate SAPs, or influence the gene expression that may refine neuronal connections [26]–[30]. 4-AP-sensitive currents, presumably of A-type K^+^ currents (I_A_) regulate spike timing and firing frequency in neurons [31]–[33] and cardiac myocytes [34]. The unique properties of the underlying channels include rapid, transient activation in the sub-threshold potentials, followed by fast inactivation. Although I_A_ is not known to be Ca^2+^-sensitive, in neurons and myocytes, the kinetics and expression have been associated with changes in Ca^2+^_i_ via channel protein interactions with Ca^2+^-sensitive proteins [35]–[39]. The functional relevance of these changes in Ca^2+^ homeostasis and I_A_ expression are unknown but the correlation of the feedback between Ca^2+^_i_ and Ca^2+^-sensitive processes, such as the expression of I_A_, can have important ramifications in Ca^2+^ oscillations and wave propagation in developing HCs [40]–[43].

In order to better understand the mechanisms of these spontaneously generated action potentials, we investigated the functional expression of I_A_ and its possible sensitivity to Ca^2+^_i_ handling. Chelation of Ca^2+^_i_ shifted the activation properties of the current to more positive potentials and reduced the expression of I_A_. Our findings provided direct evidence to demonstrate that I_A_ is tightly regulated by Ca^2+^_i_, a feedback mechanism that may shape the patterning of SAPs and ultimately sculpt synaptic connections in the developing cochlea.

## Materials and Methods

### Isolation of the Chicken Basilar Papilla

The present investigation was approved in accordance with the guidelines of the Institutional Animal Care and Use Committee of University of California, Davis. The protocol number was 15544 under the institutional authorization code A3433-01. This study included chickens at different stages of embryonic development ranging from E6–E21 as well as post-hatched chickens. Fertilized eggs were incubated at 37°C in a Marsh automatic incubator (Lyon Electric). Before experiments, chicken embryos were killed and staged according to the following: from E8–E12: based on visceral arches, feather gems and eyelids: and after E12 based on the length of the beak [44]. Basilar papillae were isolated as described previously [9]. The preparations were dissected in oxygenated chicken saline containing (in mM) 155 NaCl, 6 KCl, 4 CaCl_2_, 2 MgCl_2_, 5 Hepes, and 3 glucose, pH 7.4. The tegmentum vasculosum and the tectorial membrane were removed without any prior enzymatic treatment using a fine minutia needle. Chicken basilar papillae were stored in a 37°C incubator in Minimum Essential Medium (Invitrogen) before recording from HCs *in situ*. All experiments were performed at room temperature (21–23°C) within 5–45 mins of isolation. All reagents were obtained from Sigma Chemicals, unless specified otherwise.

### Electrophysiology

K^+^ currents were recorded in a whole-cell voltage-clamp configuration, using 2–3 MΩ resistance pipettes. Currents were amplified with an Axopatch 200B amplifier (Molecular Devices, Union City, CA) and filtered at a frequency of 2–5 kHz through a low-pass Bessel filter. The data was digitized at 5–500 kHz using an analog-to-digital converter (Digidata 1200; Molecular Devices). The sampling frequency was determined by the protocols used. No online leak current subtraction was made, and only recordings with holding currents less than 50 pA were accepted for analyses. The liquid junction potentials were measured (3.5±0.9 mV, n = 149) and corrected online [45]. The capacitative transients were used to estimate the capacitance of the cell as an indirect measure of cell size. Membrane capacitance was calculated by dividing the area under the transient current in response to a voltage step as described [9]. The capacitative decay was fitted with a single exponential curve to determine the membrane time constant. Series resistance was estimated from the membrane time constant, given its capacitance. This study included ∼703 cells with a series resistance (Rs) within a 5–15 MΩ range. After 60–90% compensation of the mean residual, uncompensated Rs was 5.1±0.5 MΩ. The seal resistance was typically 5–20 GΩ.

Action potentials were amplified (100×) with an Axopatch 200B amplifier (Axon Instruments) and filtered at 2–5 kHz through a low-pass Bessel filter. The data were digitized at 5–500 kHz using an analog-to-digital converter (Digidata 1200; Axon Instruments). The sampling frequency was determined by the protocols used. Action potentials were recorded using extracellular solution containing (in mM) NaCl 145, KCl 6, MgCl_2_ 1, CaCl_2_ 0–2, D-glucose 10, Hepes 10, pH 7.3. For whole-cell recordings of action potentials we used pipette solution containing (in mM): KCl 130, Hepes 10, D-glucose, 5 KATP, 2–10, EGTA or BAPTA. Regarding perforated patch experiments, the tips of the pipettes were filled with the internal solution containing (in mM): KCl 150, Hepes 10, D-glucose 10, pH 7.3. The pipettes were front-filled with the internal solution and back-filled with the same solution containing 250-µg/ml amphotericin. The stock solutions of all toxins were made either in ddH2O or DMSO and stored at −20°C.

To record I_A_, we eliminated Ca^2+^ from the bath and used 20 mM TEA and 300 nM apamin to block K^+^ currents, such as the delayed rectifier and Ca^2+^-activated K^+^ currents (I_K(Ca)_). Extracellular solution contained (in mM) NaCl 125, KCl 6, CaCl_2_ 0, 20 TEA, D-glucose 10, MgCl_2_ 1, HEPES 10, pH 7.3, 310 mOsm. Intracellular solution contained (in mM) KCl 120, Na_2_ATP 5, MgCl_2_ 2, HEPES 10, EGTA 1–10, or BAPTA 1–10, D-glucose 10, pH 7.3, 300 mOsm.

### Data analysis

The number of cells (n) is given for each data set. Data were analyzed using pClamp8 (Molecular Devices), Origin7.0 (OriginLab Corp. Northampton, MA) and Excel (Microsoft). Time constants (τs) were obtained from fits using Origin software. Time constants were obtained by fitting multiple exponential terms to the activation and decay of the current. The equation was of the form:
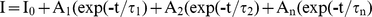
Where I_0_ is the initial current magnitude, τ_1_, τ_2_…τ_n_ are the time constants, and A_1_, A_2_…A_n_, are the proportionality constants. Voltage-dependence of activation was examined from peak amplitude (measured at ∼3–5 ms from the onset of voltage step) of tail currents at different developmental stages (embryonic, E) day-six to postnatal (P) day-two (E6-P2), then normalized steady-state curves were fitted with the Boltzmann distribution. Additionally, the steady-state inactivation curve was generated from normalized currents measured at a test potential following several conditioning pre-pulses. Pooled data were presented as mean ± SD. Significant differences between groups were tested using the Student's *t* test, with *p*<0.05 or 0.01 indicating statistical differences.

## Results

### Pharmacological elimination of I_A_ profoundly alters the structure of SAPs in developing hair cells

**Figure 1:**
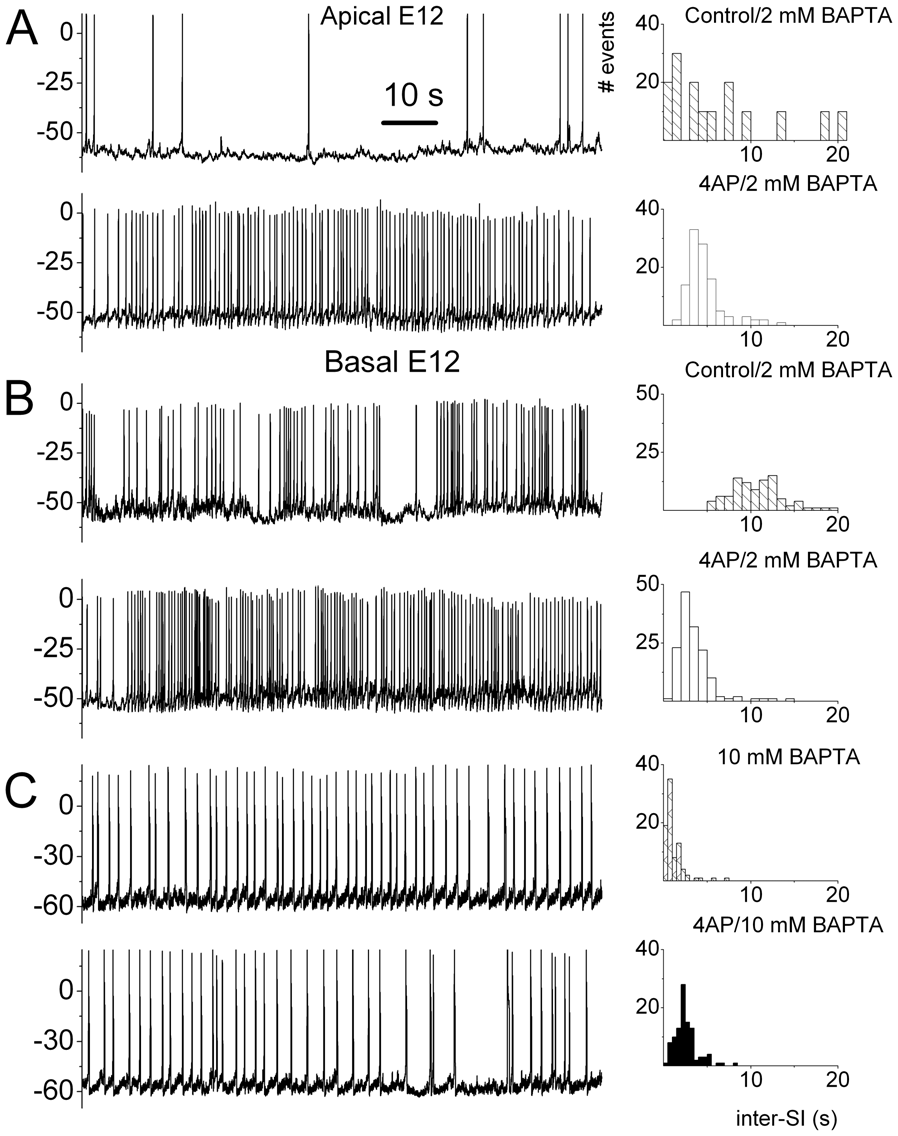
The pattern of spontaneous electrical activity in developing HCs is altered by I_A_ blocker and changes in Ca^2+^_I_. (**A**) Spontaneous electrical activity recorded from apical E12 HCs using 2-mM pipette BAPTA, for 80 s before (upper trace) and after (lower trace) application of 2.5 mM 4-AP. The interspike interval distribution histogram is shown beside (right) the recorded spontaneous action potentials (SAPs). (**B**) SAPs recorded from basal HCs at E12 using 2 mM BAPTA as in (**A**) for 80 s before (upper trace) and after (lower trace) bath application of 2.5 mM 4-AP. (**C**) SAPs recorded from a basal E12 HC using 10-mM pipette BAPTA for 80 s before (upper trace) and after (lower trace) addition of 2.5 mM 4-AP. Inhibition of the 4-AP-sensitve current promoted tonic firing. Moreover, the inhibitory effect of the 4-AP was attenuated using 10-mM pipette BAPTA. Thus, I_A_ contributes towards the patterning of SAPs in developing HCs, and this effect may be Ca^2+^-dependent.

**Figure 2:**
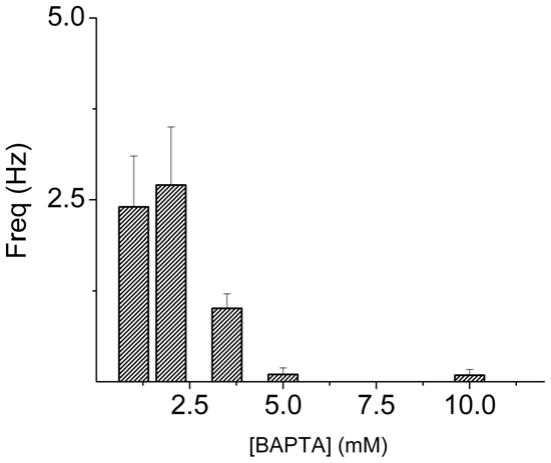
Changes in firing frequency versus intracellular BAPTA. Summary histogram illustrating the changes in firing frequency with changes in pipette BAPTA concentrations. On average, the firing frequency declined as the concentration of BAPTA was increased.

To examine the roles of I_A_ on SAPs in developing HCs, we employed pharmacological strategies [9], [46]. We applied 2.5 mM 4-AP, voltage-dependent K^+^-current (I_A_) blocker, to spontaneously active HCs from the developing basilar papilla. Shown in Figures 1A and B are the effects of 4-AP on the patterning of SAPs. An immediate observation is that 4-AP induces marked alterations in the frequency of AP firing, which is reflected in a patent reduction in the inter-spike intervals plotted on the right panels (Fig. 1). For example, 4-AP induced a 10-fold increase in AP firing in developing HCs at the apical aspects of the basilar papilla at E12 (pre-4-AP: 0.11±0.05 Hz; post-4-AP: 1.1±0.2 Hz, *p*<0.01; n = 7). Similarly, basal HCs at E12 fire APs at the rate of ∼0.5 Hz (0.5±0.2 Hz, n = 9). Moreover, after application of 4-AP, the frequency of firing increased to ∼2 Hz (2.1±0.6 Hz, n = 9; *p*<0.05). These experiments were performed using 2 mM BAPTA in the pipette solution to mimic physiological buffering capacity [47]. Spontaneous action potentials in developing HCs are Ca^2+^-dependent [9], and to assess the role of intracellular Ca^2+^ (Ca^2+^_i_) in 4-AP-mediated changes in AP firing rates, we used 10 mM pipette-BAPTA. Surprisingly, the typical phasic and burst pattern seen in E12 apical HCs (Fig. 1A) was replaced with tonic AP firing in the presence of 10 mM BAPTA (Fig. 1C). Even more startlingly was the resulting lack of 4-AP-mediated changes in the presence of 10 mM BAPTA. Changes in frequency of firing *versus* pipette-BAPTA concentrations are shown (Fig. 2). The correlation between 4-AP-mediated effects and high concentrations of BAPTA suggests that the 4-AP-sensitive current (I_A_) may be sensitive to Ca^2+^_I_ availability. Additionally, the data hinted that I_A_ regulates the firing pattern of developing HCs in a Ca^2+^_I_-dependent manner. The effects of BAPTA and 4-AP on individual action potentials are outlined in Table 1.

**Table 1:** Summary data on the effects of position and intracellular Ca^+2^ buffering on spike width, and rate of change of voltage of the depolarizing and repolarizing phase of spikes at E12.

	Max left slope (dV/dt, V/s)		Max right slope (dV/dt, V/s)		Half width (ms)	
	Control	4-AP	Control	4-AP	Control	4-AP
Apical 2 mM BAPTA	6.9±0.7	2.9±1.9	6.4±0.6	4.2±1.8	6.3±0.4	4.4±1.5
Basal 2 mM BAPTA	5.9±0.3	5.9±0.8	5.9±0.2	5.8±0.7	5.9±0.2	4.2±2.6
Basal 10 mM BAPTA	15.9±1.2	15.6±1.1	5.9±0.7	5.9±0.7	9.9±1.5	10.1±1.5

Increase in intracellular buffering significantly affected the spike width, and rate of change of voltage of the depolarizing and repolarizing phases of spikes. The data is expressed as mean ± SD (n = 9 cells for each experiment).

**Figure 3:**
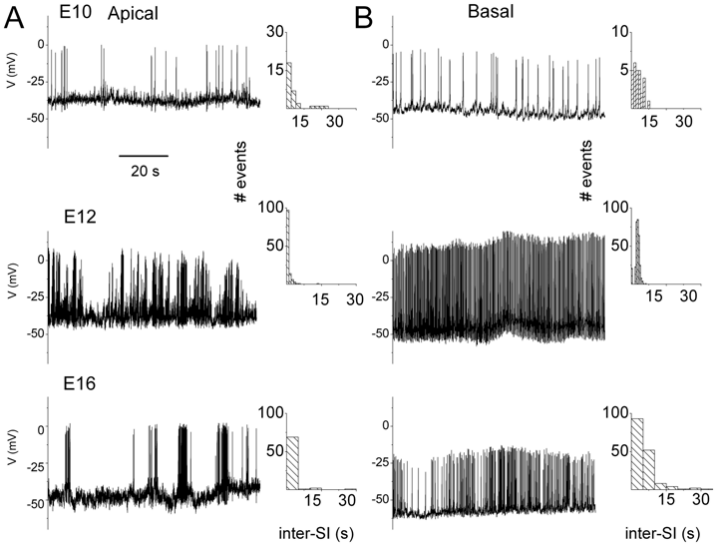
Changes in the structure of spontaneous electrical activity in developing HCs. Examples of the spontaneous activity recorded using perforated patch from apical (**A**) and basal (**B**) aspects of the developing chicken basilar papilla at E10, E12 and E16. The inter-spike interval distributions are shown.

The structure of SAPs in developing HCs not only differs between apical and basal cells, but also varies at different developmental stages as shown in figure 3. Along the tonotopic axes, the general trend varied from phasic bursting apical cells to tonic bursting basal cells (Fig. 3). Thus, we reasoned that different kinetics and the magnitude of I_A_ during development, and tonotopic expression of the current could, in part, be responsible for diverse spiking activity (Fig. 3).

### I_A_ is expressed at the earliest stages of HC development

**Figure 4:**
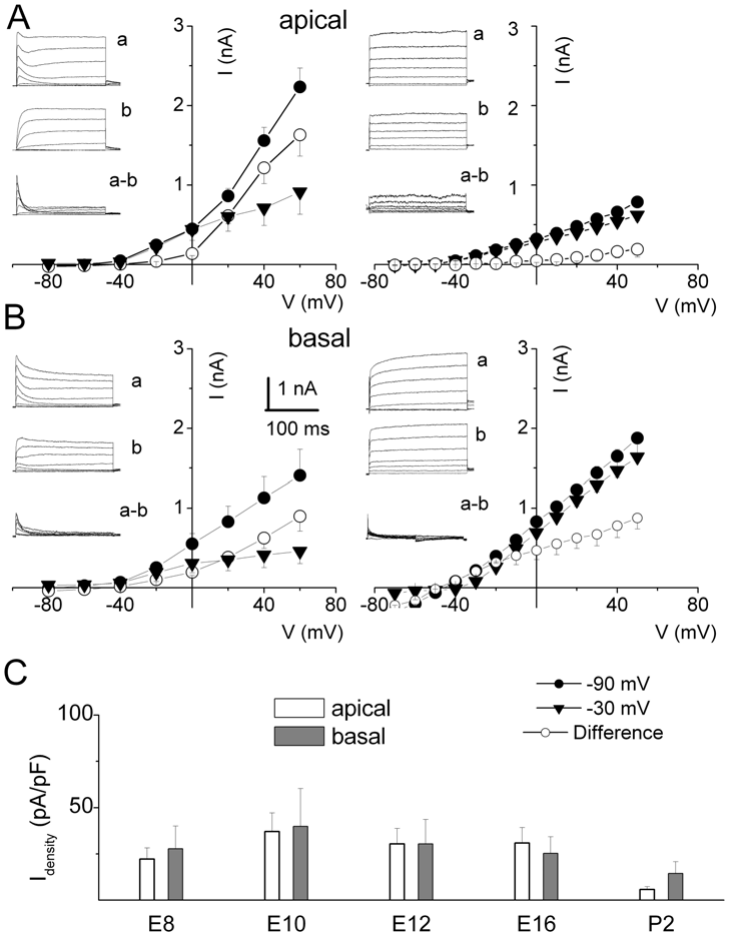
The transient K^+^ currents displayed sensitivity towards holding potentials, and the magnitude changes during development. (**A–B**) Examples of current traces recorded at E12 and P2 from HCs from apical and basal aspects of the basilar papilla. Current traces were elicited using 250-ms depolarizing voltage steps in 10-mV increments from −80 to 50 mV. For clarity, traces are shown in 20 mV increments. The holding potentials were −90 mV (*a*) and −30 mV (*b*). The difference in current (*a–b*) is shown as well. (**A–B**) Plots of the mean current-voltage relationships are shown, corresponding to changes in the magnitude of the transient current at different developmental stages. (**C**) Mean difference current density at +0 mV at apical and basal regions of the basilar papilla (pA/pF) at E8, E10, E12, E16, and P2. The holding potential-sensitive current density decreased during development. (E8, n = 4; E10, n = 7; E12, n = 16; E16, n = 5; P2, n = 9).

**Figure 5:**
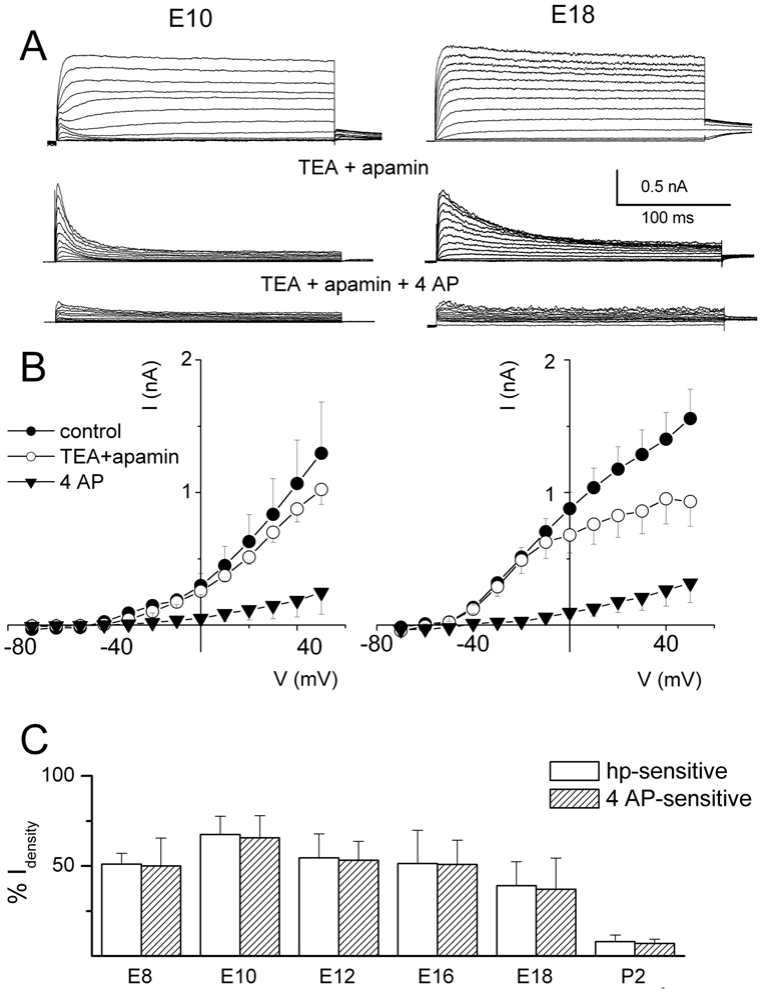
Holding potential-sensitive K^+^ currents are sensitive to 4-AP. (**A**) An example of whole cell current recorded from apical HCs at E12 and E18 showing TEA and 4-AP-sensitive components, using the protocol described in Figure 1. (**B**) Mean peak current-voltage relationships are plotted below the traces. (E12, n = 11; E18, n = 5). (**C**) Histogram showing the holding potential-sensitive currents was similar in magnitude to the 4-AP-sensitive currents. Additionally, the magnitude of the holding potential- and 4-AP-sensitive current decreased with maturation (n = 6).

Previous studies have shown that K_v_4.2 and K_v_1.x channels are the presumptive channels that generate I_A_ in chicken HCs. These channels were expressed, at the level of mRNA, at the earliest stages of development [48]{Sokolowski, 2004 #192, [49]}. However, functional studies in mature HCs showed that the current was limited to basal HCs [50], [51]. We determined the functional expression and kinetics of I_A_ in HCs with respect to developmental age and position along the tonotopic axis. Figure 4 shows examples of whole-cell current profiles and current-voltage relations in HCs at the apical (Fig. 4A) and basal (Fig. 4B) aspects of the basilar papilla elicited from different holding potentials at E8-P2 (Fig. 4C; summary data). The transient component of the current was remarkably sensitive to holding voltages throughout development. The difference-current between current traces generated at a holding potential of −90 and −30 mV was the main transient component. The magnitude of the transient current plummeted as HCs matured (Fig. 4C), and the transient outward current was virtually absent in mature apical HCs. To ensure that the transient current was indeed I_A_, we examined the sensitivity of the current to 4-AP [50], [51]. A sizable portion (∼50% at E10) of the sustained component of the outward K^+^ current elicited from a holding potential of −90 mV was suppressed upon application of 20 mM TEA and 300 nM apamin in the bath, revealing a fast inactivating current that was sensitive to 2.5 mM 4-AP (Fig. 5A). Figure 5B illustrates the current-voltage relations at E10 and E18, and figure 5C shows the summary data, revealing a striking agreement between the magnitudes of the 4-AP- and holding potential-sensitive currents across different developmental stages of basal HCs. The result is in keeping with molecular biological evidence showing the expression of K_v_4.2 channels in developing HCs [48].

### Changes in voltage-dependent activation and inactivation of I_A_ during development

**Figure 6:**
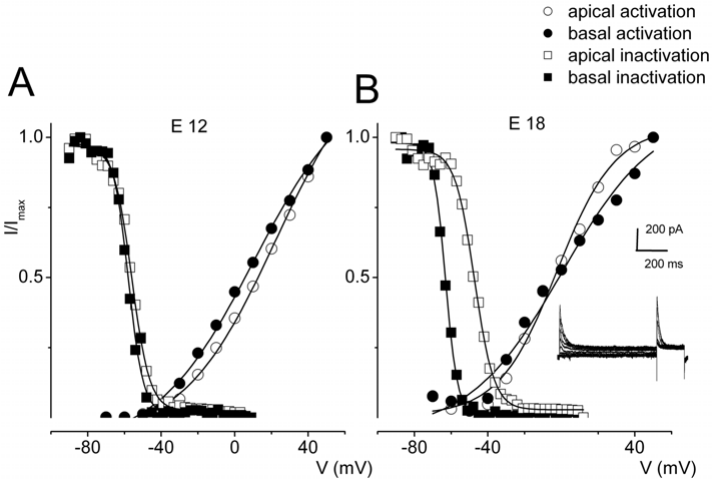
Voltage dependence of activation and inactivation of I_A_ changes with maturation of HCs. **A–B** The voltage-dependence of activation and inactivation of the transient current from HCs at apical and basal aspects of the basilar papilla at E12 and E18 were assessed. Steady-state inactivation properties of the transient current were determined by presenting pre-pulses of ∼1-sec duration, at different membrane potentials (−90 to −10 mV), followed by a test pulse at 0 mV for 300 ms (see inset for examples of current traces). The Boltzmann function fits for steady-state activation and inactivation are plotted with solid lines. The half activation (V_1/2_) and inactivation voltages as well as slope factors are summarized in Tables 2, 3, 4.

The voltage-dependence of the activation and inactivation of I_A_ varied with age (Figure 6, Tables 2, 3, 4). Differences in voltage-dependent properties of I_A_ between apical and basal HCs were stark at E18 compared to E12 (Fig. 6B). At E12, the V_1/2_ of the activation curve was ∼0.5 mV for apical and ∼−5.5 mV for basal cells. The half-activation voltage (V_1/2_) of inactivation was ∼−56 mV for apical and ∼−59 mV for basal cells. By E18, the transient outward current in basal HCs inactivated at more negative potentials than apical HCs (Figure 5B; Table 2). At E18, the V_1/2_ of inactivation was ∼−47 mV for apical and −63 mV for basal cells. Consistent with previous reports, the data suggest that as HCs mature, I_A_ is mainly confined to cells at the basal aspects of the cochlea [50]–[52].

**Table 2:** Summary data on the effects of development and tonotopic position of HC-A-current activation and inactivation (V_1/2_ (mV), k (mV)) with 2 mM BAPTA in pipette solution.

	Activation				Inactivation			
	Apical		Basal		Apical		Basal	
Age	V_1/2_	k	V_1/2_	k	V_1/2_	k	V_1/2_	k
E12	0.5±1.1	25.4±2.5	−5.5±3.6	26.6±3.2	−56.0±0.2	4.9±0.2	−59.2±0.9;	4.8±0.9
E18	−0.5±2.1	25.5±7.3	−2.4±2.1	15.8±2.1	−47.3±0.3	5.2±0.3	−63.0±0.2;	3.3±0.2

Although there is a small difference in voltage dependence of inactivation between apical and basal region of the papilla, this difference becomes significant at more mature stages (n = 7 cells).

**Table 3:** Voltage dependence of activation (V_1/2_ (mV), k (mV)).

	1 mM BAPTA		2 mM BAPTA		5 mM BAPTA		10 mM BAPTA		5 mM EGTA		10 mM EGTA	
Age	V_1/2_	k	V_1/2_	k	V_1/2_	k	V_1/2_	k	V_1/2_	k	V_1/2_	k
E12	0.6±1.9	22.8±1.4	0.5±1.1	25.4±2.5	8.9±2.6	25.1±1.8	23.1±5.7	24.5±1.9	−0.8±0.4	16.3±2.5	23.3±4.3	25.3±2.6
E18	−3±0.9	22.1±0.9	−10.1±0.8	16.9±0.9	7.1±1.4	22.9±1.0	6.5±0.7	20.9±0.6	-		-	

The table compares the voltage dependence of the steady state activation at different stages of development and intracellular Ca^2+^ buffering. Increased buffering shifts the activation to more positive potentials (n = 8 cells).

**Table 4:** Voltage dependence of inactivation (V_1/2_ (mV), k (mV)).

Age	1 mM BAPTA		2 mM BAPTA		5 mM BAPTA		10 mM BAPTA	
	V_1/2_	k	V_1/2_	k	V_1/2_	k	V_1/2_	k
E12	−50.1±2.0	0.50±0.1	−56.1±0.2	4.8±0.2 (a)	−52.3±0.2	3.9±0.2	−48.4±0.5	2.6±0.7(a)
	−57.5±0.3	4.3±0.3	−59.2±0.9	8.1±0.9 (b)			−55.6±0.7	9.2±0.6(b)
E16	-		−59.1±2.1	4.1±2.2	−57.1±0.1	3.3±0.1	−52.5±0.7	2.2±0.5(a)
							53.4±8.1	1.5±3.5(b)
E18	-		−55.7±0.3;	4.2±0.3 (a)	−46.2±0.7	3.3±0.4 (a)	−48.9±1.4	1.6±1.2(a)
			−63.1±0.2	3.4±0.2 (b)			−47.4±0.4	5.2±0.4 (b)

The table illustrates a comparison of voltage dependence of steady-state inactivation at different stages of development and the concentrations of intracellular Ca^2+^ buffer. Increased buffering shifts the activation to more positive potential (a = apical, b = basal hair cells; n = 7).

### Changes in Ca^2+^_I_ buffering have a profound effect on I_A_

**Figure 7:**
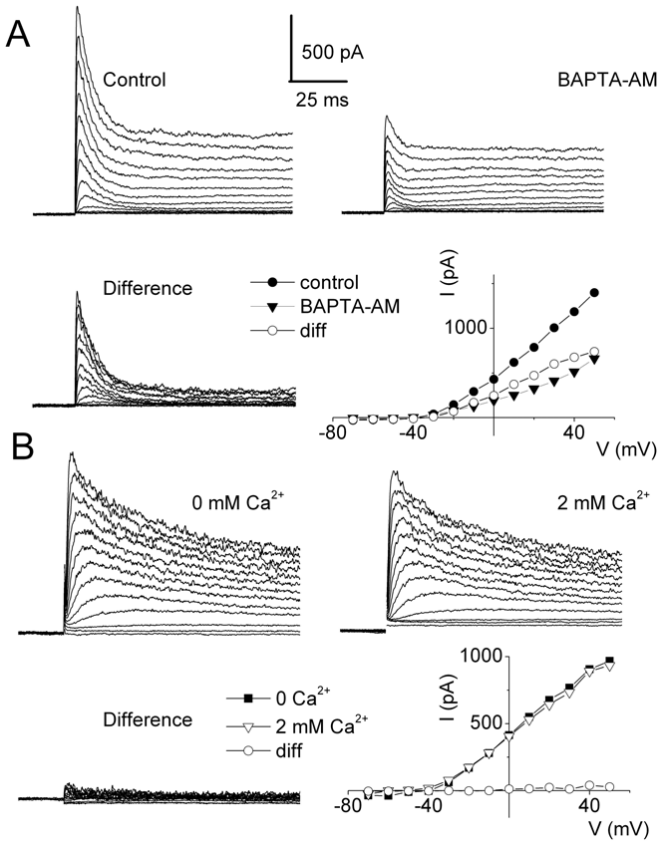
I_A_ is sensitivity to Ca^2+^_I_ buffering. (**A**) Examples of current traces recorded from different apical HCs at E14 using 1 mM pipette BAPTA in the presence of 20 mM TEA and 200 nM apamin in the bath solution before and 30 mins after application of bath solution containing 5 mM BAPTA-AM. The difference-current traces are shown. An increase in Ca^2+^_I_ buffering altered the transient K^+^ current. The current-voltage relationship is shown. (**B**) Examples of currents recorded from apical HCs at E14 in the presence of 20 mM TEA and 200 nM apamin before and after application of a bath solution containing 2 mM Ca^2+^, as well as the difference current. The currents were elicited with the same protocol as described in Figure 1. The plot shows the current-voltage relationship.

**Figure 8:**
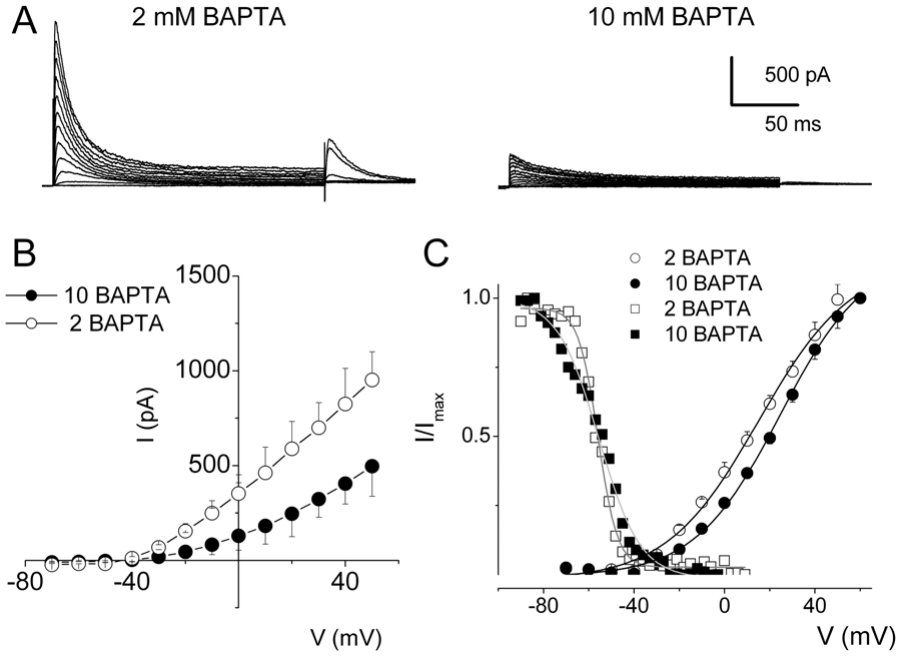
The voltage-dependent activation and inactivation of I_A_ and availability of Ca^2+^_I_. (**A**) Current traces recorded from apical HCs at E12 using pipette BAPTA concentrations, 2 (left panel) and 10 mM (right panel) BAPTA. (**B**) Mean current-voltage relationship for data obtained for the two conditions (2 and 10 mM pipette BAPTA; n = 9). (**C**) The voltage-dependence of activation and inactivation of the transient K^+^ current from E12 apical HCs using 2 and 10 mM pipette BAPTA. Steady state inactivation properties of the transient current were determined using the protocol described in Figure 6. Steady-state activation and inactivation of the currents were determined as in Figure 6. The Boltzmann function fits are shown in solid lines. Half-activation voltages were (in mV) 0.5±1.1 and 33.1±5.7 for 2, and 10 mM BAPTA, respectively. The maximum slope factors (k) were (in mV): 25.4±2.5 and 24.5±1.9 (n = 9 cells) for 2 and 10 mM BAPTA, respectively. The half-inactivation voltages were (in mV) −56.1±0.2 and −48.4±0.5 for 2, and 10 mM BAPTA, respectively. The maximum slope factors (k) were 4.8±0.2 and 2.6±0.7 (n = 9 cells) for 2, and 10 mM BAPTA, respectively. Notice the currents' activation and inactivation is shifted to more positive potentials with increased Ca^2+^ buffering (see Tables 4).

**Figure 9:**
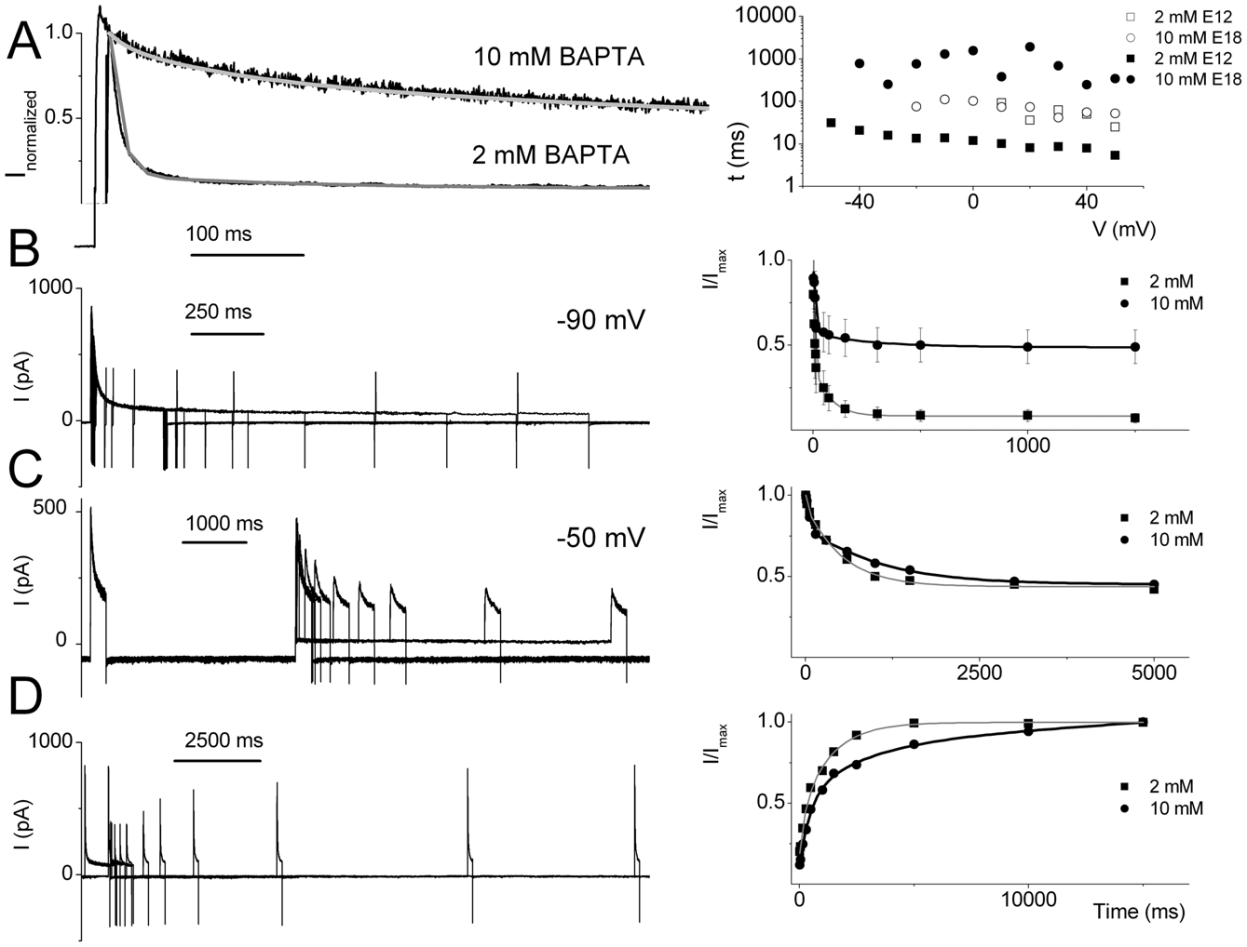
Kinetics of inactivation of I_A_ in the developing HCs is affected by Ca^2+^_I_ availability. (A) Normalized current traces elicited using ∼1 s pulse for E12 cells using 2 mM (dark gray line) and 10 mM BAPTA (light gray line) in the pipette (Left). The fast components of decay were eliminated with increased BAPTA as well as with maturation. Holding potential was −90 mV and voltage steps were in 10 mV increments. Current decay could be best fitted with two different time constants (τ) at all stages of development. The time constants were prolonged with an increase in intracellular BAPTA as well as with maturation [τs (in ms) for E12, using 2 mM BAPTA were: 5±3, and 75±13 (n = 11 cells); τs for 10 mM BAPTA were 67±3, and 498±13 (n = 10 cells) and for E18: 52±13, and 611±25 (n = 7 cells)]. For the example shown at E12, the exponential fits yielded τ_1_ = 5 ms and τ_2_ = 70 ms for 2 mM BAPTA. The current traces illustrated from the E18 HCs were fitted with τ_1_ = 50 ms and τ_2_ = 600 ms. See Table 4 of summary data. (B) The time course of the development of inactivation at −90 mV (Left) and the τs of development of inactivation were determined (Right) for current traces recorded at E12 for 2 mM BAPTA and 10 mM BAPTA [τs for 2 BAPTA: 5.2±2.3, 75.2±28.8 (n = 8 cells); τ for 10 BAPTA: 8.7±4.1 ms; 289±32.9 (n = 8 cells)]. (C) Examples of traces generated to examine the time course of the development of inactivation at −50 mV for E12 currents. An example of such protocol on an E12 cell is shown (Left). The exponential fits to the data are shown on the right; τs (in ms) for E12: 515±161 using 2 mM BAPTA and 997±203 ms using 10 mM BAPTA in the pipette (n = 6 cells). (D) To estimate how quickly the current recovered from inactivation, we measured recovery kinetics after 1-second at the holding potential of −90 mV, using standard recovery time protocol. We measured the amplitude of the transient potassium currents by depolarizing to a fixed potential after variable time at −90 mV. Amplitude of these currents was normalized to the amplitude of the currents activated from −90 mV and plotted against the duration of steps to −90 mV. Examples of traces generated to examine the time course of the recovery from inactivation for E12 currents (Left). The right panel shows the time course and the exponential fits [Fits in solid lines, ts for E12: 446±56 ms using 2 mM BAPTA and 1286±78 ms using 10 mM BAPTA in the pipette (n = 5 cells)]. Amplitude of the currents was normalized to the amplitude of the currents activated from a holding potential of −90 mV and plotted against the duration of step to −90 mV.

To examine the underlying mechanisms for the BAPTA-induced 4-AP-insensitivity to SAPs described in figure 1, we tested I_A_ sensitivity to acute changes in Ca^2+^ chelators. We used BAPTA-AM, a selective Ca^2+^ chelator that is the cell-permeable analog of BAPTA. Application of 5 mM BAPTA-AM to basal HCs produced a marked reduction of the transient K^+^ current (Fig. 7A). Moreover, raising the external Ca^2+^ from 0 to 2 mM had no effect on the transient current (Fig. 7B), hinting that Ca^2+^ entry via Ca^2+^ channels may not be sufficient to affect I_A_. Rather, the release of Ca^2+^_i_ may be the Ca^2+^ source to alter I_A_. We probed the Ca^2+^-dependence of I_A_ further by examining the voltage- and time-dependent properties of the current using varying concentrations of BAPTA (Tables 3, 4, 5). Figure 8 illustrates the effects of 2 and 10 mM intracellular BAPTA on I_A_ in an apical E12 HC. Reducing the available Ca^2+^_I_ by using 10 mM pipette BAPTA produced a significant shift in the voltage-sensitivity of activation. The V_1/2_s of the steady-state activation were (in mV) ∼0 and 23 using (in mM) 2 and 10 BAPTA, respectively. Moreover the V_1/2_s of the steady-state inactivation were ∼−56 mV and −48 mV using (in mM) 2 and 10 BAPTA, respectively. Additionally, we examined the time-dependence of the development of inactivation after varying durations at −50 mV and −90 mV. The time constants of inactivation (τ_I_) were compared at different stages of development and with respect to different concentrations of Ca^2+^_I_ buffer (Table 5). At all developmental stages tested (E12–E18), the kinetics of decay of the current were fitted with two τs. As shown in figure 9, the use of different concentrations of BAPTA had a marked effect on the amplitude and inactivation kinetics of I_A_. For example, for an apical E12 HC, the amplitude of the current measured at +40 mV was ∼825 pA in 2 mM BAPTA and ∼405 pA in 10 mM BAPTA. Moreover, inactivation time constants (τ_I_s) of I_A_ were ∼5 ms and 75 ms, using 2 mM BAPTA. Using 10 mM BAPTA the τs were ∼75 ms and 300 ms (Fig. 9A). Similarly, the time dependence of development and recovery from inactivation were duly affected (Figs. 9B–D). Whereas the current shows complete inactivation within ∼75 ms, using 2 mM BAPTA, the time constant of inactivation was prolonged by ∼ 4-fold (∼300 ms) in the presence of 10 mM BAPTA (Figure 9C). Meanwhile, the current recovered from inactivation with a time constant of ∼450 ms in 2 mM BAPTA and ∼1200 ms in 10 mM BAPTA (Fig. 9D). The results clearly suggest that the 4-AP-sensitive current is regulated tightly by Ca^2+^_i_.

**Table 5:** Comparison of the time constants (τ) of decay at different stages of development and with respect to different concentrations of Ca^2+^_I_ buffer.

Age	2 mM BAPTA	τ (ms)	10 mM BAPTA	τ (ms)
	τ	τ_2_	τ_1_	τ_2_
E12	5	75	60	298
E16	30	250	40	230
E18	50	600	50	700

At all developmental stages tested (E12–E18), the kinetics of decay of the current were fitted with two time constants.

## Discussion

The transient outward K^+^ current (I_A_) in the developing chicken HC was identified functionally by Murrow (1994) and Griguer and Fuchs (1996) [51], [53] and the molecular characteristics were revealed later [48] {Rajeevan, 1999 #193, [52]}. Moreover, the short and long-term modulation of I_A_ in developing chicken HC is unknown, despite the currents purported role in spike timing in SAPs. Also important, roles of I_A_ and its regulation and the ensuing effects on the structure of SAPs in developing HCs are unclear. This study fills in major gaps in our understanding of the patterning of SAPs in HCs. Our findings include; 1) I_A_ contributes towards the patterning of SAPs. Since the current dictates the spike timing and firing pattern, it is conceivable to suggest that it may regulate the release of neurotransmitters in the developing cochlea to sculpt synapse formation. 2) Expression of I_A_ is regulated developmentally and tonotopically. At the apical aspects of the basilar papilla the expression of I_A_ is prominent only in early development. By contrast, I_A_ persists in HCs at the basal aspects of the matured basilar papilla. 3) Alterations of Ca^2+^_I_ by Ca^2+^ buffers produced marked effects on the functional expression, voltage-dependence and kinetics of I_A_. Increased concentrations of the Ca^2+^_I_ buffer, BAPTA shifted the voltage-dependence of activation and inactivation to more positive potentials and reduced the number of functional channels expressed. These findings reaffirm the tight interplay that occurs between Ca^2+^_I_, and K^+^ currents.

Suppression of I_A_ visibly alters spike timing and frequency, transforming phasic action potential burst into tonic firing in spontaneously active chicken HCs. The results are reminiscent of the effects of a transient outward current in the developing mouse HC [16]. However, after increased Ca^2+^ buffer, not only did the phasic patterning of spikes appear tonic, but also suppression of I_A_ had no effect on the firing pattern, raising the possibility that reduced Ca^2+^_I_ could stifle the availability of I_A_. The deduced amino acid sequence of the α-subunits of K_v_1.2, K_v_1.3 and K_v_1.5 and the auxiliary subunit, K_v_β1 that confer A-type transient current reveals multiple putative phosphorylation sites at the N- and C-termini [48] {Rajeevan, 1999 #193 [52], [54]}. Indeed, several of these potential regulatory sites and the second messengers involved have been demonstrated to be Ca^2+^-dependent. For example, there is a Src tyrosine kinase proline-rich binding site at the N-terminal of K_v_1.5 that confers Ca^2+^-dependent modulation of the kinetics and voltage-dependent activation of transient K^+^ currents in Schwann cells [55], [56]. Alternatively, it is conceivable that the presences of multiple potential Ca^2+^-dependent regulatory sites at the C-terminus of the channel and other interacting partners that form the channel complex could affect the localization, expression and specific functional properties of the native K^+^ conductance, as observed in cardiac myocytes and neurons [57]–[63].

Ca^2+^ handling in the developing chicken basilar papilla may undergo marked plasticity to account for varying demands for the expression of Ca^2+^-dependent processes and increased Ca^2+^ influx through increased transduction channel numbers [64]. For example the expression of the mobile Ca^2+^ buffer, calbindin, is regulated during development and along the tonotopic axis of the cochlear in accordance with the demands' of Ca^2+^ regulation [64]. Similar assortments of Ca^2+^ regulation can be seen in the apico-basal gradient of the basilar papilla [65]. The differential and increased expression of Ca^2+^ buffers during development dovetails well with the expression pattern of Ca^2+^ buffers [64]. In keeping with the expression pattern of Ca^2+^ buffers in HCs of the developing basilar papilla and our findings, the kinetics and voltage-dependence of I_A_ are expected to be altered. Voltage dependence of the activation and inactivation of I_A_ are shifted to more positive potentials with increased Ca^2+^ buffers. Throughout development, basal HCs express I_A_ of faster kinetics of inactivation than at the apical aspects of the basilar papilla. Moreover, with maturation this difference is magnified as apical HCs lose the transient component of K^+^ currents. At the basal aspects, the ratio of I_A_ to the total outward K^+^ current is diminished with maturation. Whereas these findings are in accordance with changes in Ca^2+^ handling in the chicken basilar papilla, they are in stark contrast to reports on the expression of 4-AP sensitive current, presumably I_A_, in the developing mouse HC, where the expression of the current increases with development and persists in mature HCs [16], [66]. Together, our data demonstrates that reduction of Ca^2+^_I_ availability has profound effects not only on the kinetics of I_A_ and the number of channels expressed in HCs, but also on the ensuing SAP, which is sculpted by the current.
