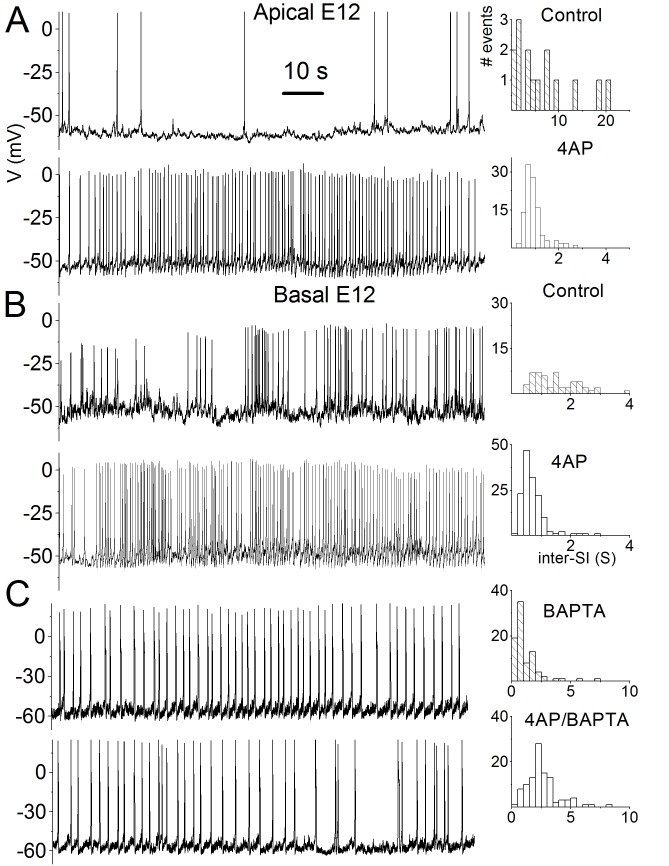# Correction: The Activity of Spontaneous Action Potentials in Developing Hair Cells Is Regulated by Ca^2+^-Dependence of a Transient K^+^ Current

**DOI:** 10.1371/annotation/f68dd05a-c636-49b3-8869-af7815c6a997

**Published:** 2013-10-30

**Authors:** Snezana Levic, Ping Lv, Ebenezer N. Yamoah

A representative trace is present in error. Please see the correct Figure 1 here: 

**Figure:**